# Towards “bionic” proteins: replacement of continuous sequences from HIF-1α with proteomimetics to create functional p300 binding HIF-1α mimics†
†Electronic supplementary information (ESI) available: Detailed experimental procedures and characterisation; additional biophysical data. See DOI: 10.1039/c6cc01812b



**DOI:** 10.1039/c6cc01812b

**Published:** 2016-03-24

**Authors:** George M. Burslem, Hannah F. Kyle, Alexander L. Breeze, Thomas A. Edwards, Adam Nelson, Stuart L. Warriner, Andrew J. Wilson

**Affiliations:** a School of Chemistry , University of Leeds , Woodhouse Lane , Leeds , LS29JT , UK; b Astbury Centre for Structural Molecular Biology , University of Leeds , Woodhouse Lane , Leeds , LS29JT , UK . Email: a.j.wilson@leeds.ac.uk; c School of Molecular and Cellular Biology , Faculty of Biological Sciences , University of Leeds , Woodhouse Lane , Leeds LS2 9JT , UK; d Discovery Sciences , AstraZeneca R&D , Alderley Park , Cheshire , SK10 4TG , UK

## Abstract

Using the HIF-1α transcription factor as a model, this manuscript illustrates how an extended sequence of α-amino acids in a polypeptide can be replaced with a non-natural topographical mimic of an α-helix comprised from an aromatic oligoamide. The resultant hybrid is capable of reproducing the molecular recognition profile of the p300 binding sequence of HIF-1α from which it is derived.

## Abstract 2

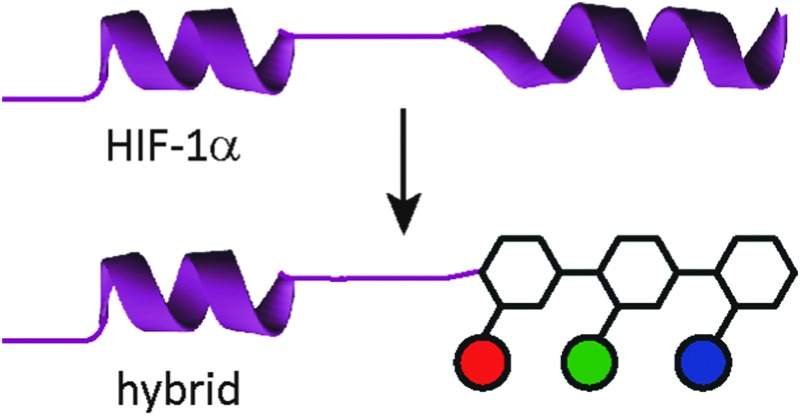
An extended sequence of α-amino acids in HIF-1α is replaced with a non-natural topographical mimic of an α-helix comprised from an aromatic oligoamide to reproduce its p300 recognition properties.

Nature uses a stunning array of architectures to carry out complex tasks including catalysis and cell signalling. Biopolymers are able to perform these feats because they self-organise and present functional motifs (*e.g.* an active site or binding surface) through precise 3-D orientation of primary structure. A long term goal in chemical and synthetic biology, therefore, is to expand on this diversity, and through the incorporation of non-natural functionality, elaborate bio-macromolecules with enhanced or orthogonal functionality and/or properties.^1^ Foldamers are oligomers that adopt well-defined conformations and either replicate structural and functional features of natural biomacromolecules or access, using non-natural building blocks, novel folds and functions not found in nature. An alternative approach to this bottom-up strategy, termed “protein-prosthesis”, lies at the cross-roads with strategies to chemically^2–4^ or genetically^5^ introduce non-natural function into proteins. In protein prosthesis,^6,7^ backbone engineering^8^ allows individual residues or sequences within proteins to be replaced with non-natural residues.^9–13^ Notable examples include the incorporation of β-amino acid residues in the B1 domain of *Streptococcal* protein G (GB1)^14^ and Betabellin-14,^15^ the re-engineering of a heterodimeric chorismate mutase enzyme^16^ using sequence based design, the replacement of loop regions in GB1 using PEG^17^ and the incorporation of an entire β-amino acid topological mimic of an α-helix into IL8.^18^ α-Helix mimetics^19–23^ employ a suitably functionalised generic scaffold to reproduce the spatial projection and composition of key side chains found at a helical interface between two proteins. Such α-helix mimetics have been shown by us^24,25^ and others^26–28^ to act as potent inhibitors of protein–protein interactions, but there are limited studies on their incorporation into higher-order structures.^29^ In this manuscript we illustrate the first steps towards this goal by replacement of an entire segment of the HIF-1α transactivation domain with an aromatic oligoamide (Fig. 1). In doing so, we provide the first example of a replacement of an extended peptide sequence with a topographical mimic.

**Fig. 1:**
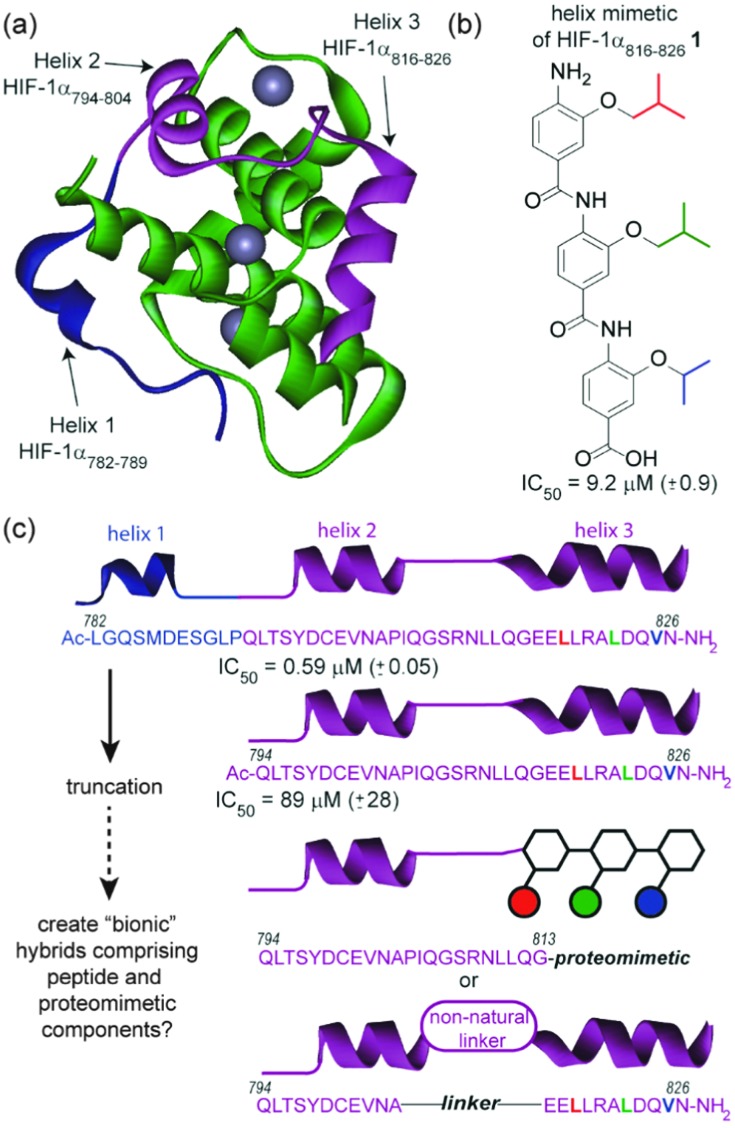
Approach for assembly of a “bionic” protein (a) NMR structure of the HIF-1α C-TAD in complex with p300; PDB ID: 18LC (p300 in green, helix 1 of HIF-1α in blue, key binding sequence comprising helices 2 and 3 of HIF-1α in purple and zinc as grey spheres), (b) structure of a proteomimetic inhibitor of HIF-1α/p300 based on helix 3 of HIF-1α (*i*, *i* + 4 and *i* + 7 mimicking side chains coloured red, green and blue respectively) (c) schematic depicting prior truncation studies on HIF-1α C-TAD together with conceptual structure of hybrids comprising peptide and proteomimetic or non-natural linkers.

Our design focused upon a minimal region of the HIF-1α C-terminal transactivation domain (C-TAD) which interacts with the CREB-binding protein (CBP)/p300 co-activator to promote transcription (Fig. 1a).^30,31^ Previously our group illustrated that two helical domains (HIF-1α_794–826_, *i.e.* helix 2/3) within the 42 residue C-TAD peptide (HIF-1α_782–826_) were necessary for measurable binding to p300 (Fig. 1c).^32^ We had also identified a helix mimetic **1** based on our 3-*O*-alkylated aromatic oligoamide scaffold which was designed to mimic helix 3 of the HIF-1α C-TAD (HIF-1α_816–826_) and shown to act as a low μM (IC_50_ = 9.2 ± 0.9 μM) inhibitor of the HIF-1α/p300 interaction (Fig. 1b).^33^ In our design (hybrid **2**), the helix 3 region (HIF-1α_816–826_) of HIF-1α (HIF-1α_782–826_) was replaced with the previously identified helix mimetic^33^ and the remainder of the sequence preserved (Scheme 1a). In parallel, we also designed a series of helix 2/3 conjugates **3a–c** linked by PEG spacers (Scheme 1a); the purpose in designing these compounds was to ascertain the extent to which the p300 binding potency might be maintained when the linker region between key helical regions (HIF-1α_794–808_ and HIF-1α_816–826_) was replaced.

**Scheme 1:**
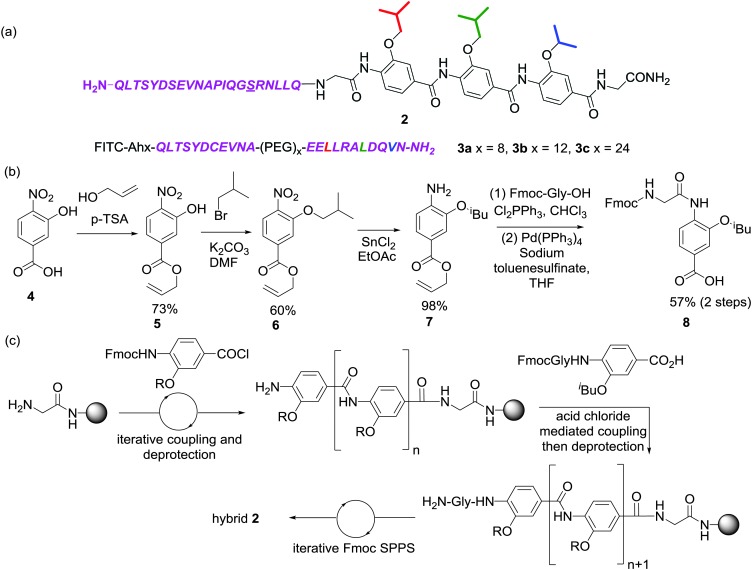
(a) Structure of peptide-helix mimetic hybrid **2** and **3a–3c** (PEG = polyethylene glycol), (b) synthetic route for assembly of the key N-terminal Fmoc glycyl 3-butyloxy-4-aminobenzoic acid building block **8** (c) outline of approach used to synthesise hybrid **2**.

Previously developed solid-phase synthesis methods for the preparation of oligobenzamides facilitate rapid preparation of helix mimetic trimers^34^ but we found the method unsuitable for preparation of the target peptide-helix mimetic conjugate **2**. To address the poor nucleophilicity of trimeric oligoamides with a terminal aniline, Fmoc-glycine was coupled to an isobutyl monomer **7** using dichlorotriphenylphosphorane (Scheme 1b). Noteworthy in this sequence was the use of an allyl ester, which facilitated ester cleavage in the presence of the base-labile Fmoc protecting group. Fischer esterification of nitro acid **4** with allyl alcohol gave ester **5** followed by alkylation with isobutyl bromide to give alkoxy nitro ester **6**. The aryl nitro group was reduced to the aniline **7** using stannous chloride then acylated with Fmoc-glycine *via in situ* acid chloride formation with dichlorotriphenylphosphorane. Finally the acid was revealed with palladium(0) tetrakis triphenylphosphine and sodium toluenesulfinate scavenger^35^ to give the final building block **8** in good yield.

The desired peptide-helix mimetic hybrid **2** was subsequently obtained using Fmoc based solid-phase synthesis (Scheme 1c). *O*-Alkylated 2-hydroxybenzoic acid monomers were assembled on resin using the previously described microwave assisted approach until Fmoc-Gly-^i^Bu dipeptide building block **8** had been coupled. Following deprotection, the oligomer was then extended using conventional Fmoc solid phase synthesis with HCTU-mediated couplings. The preparation of hybrid **2** was performed in a single run on an automated peptide synthesiser (CEM Liberty) followed by HPLC purification (see ESI† for additional details). PEG linked helix 2/3 conjugates **3a–c** were all synthesized using conventional Fmoc based solid phase synthesis and labelled with fluorescein to permit direct binding analyses (Scheme S2a, see ESI,† for details of the synthesis).

The hybrids **2** and **3a–c** were then tested in a previously described fluorescence anisotropy p300/HIF-1α competition and p300 binding assays respectively (Fig. 2a).^33^ Hybrid **2** was shown to inhibit the interaction with comparable affinity to the HIF-1α_794–826_ peptide from which it was derived (IC_50_ values hybrid **2** = 83 ± 1.8 μM; HIF-1α_794–826_ = 89 ± 2.8 μM).^32^ Crucially, to avoid disulfide formation, the helix mimetic peptide **2** conjugate bears a cysteine to serine modification (underlined in Scheme 1), whereas the peptide does not. The mutation of this cysteine residue to a serine has been shown to reduce affinity by approximately 10-fold for the native sequence,^36^ which should be considered when comparing the affinities. It should also be noted that this affinity does not derive solely from the peptidic region, as a peptide comprising the same residues (HIF-1α_794–815_) shows no inhibitory activity (IC_50_ > 500 μM).^32^

**Fig. 2:**
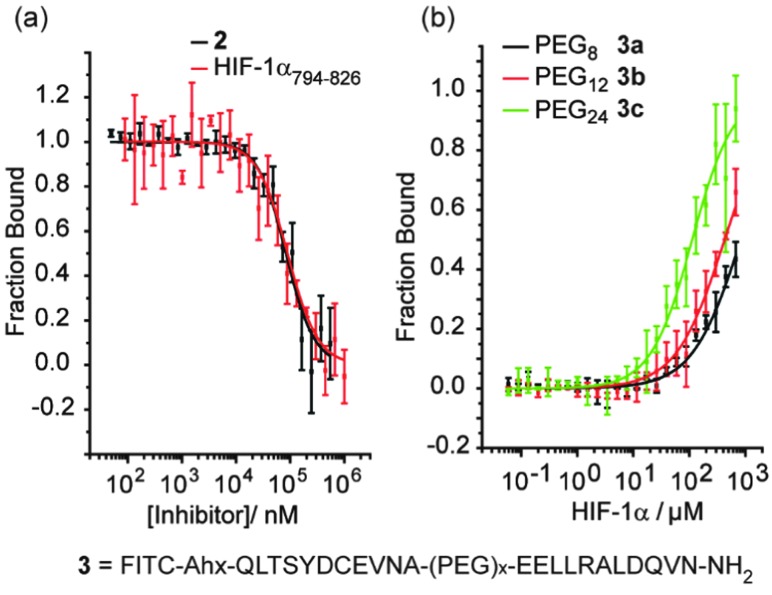
Biophysical screening of HIF-1α peptides and hybrids, HIF-1α_794–826_, **2** and **3a–c** (a) fluorescence anisotropy (FA) competition data for inhibition of p300/FITC-HIF-1α C-TAD for HIF-1α_794–826_ and **2** (b) (FA) binding data for **3a–c** to p300 (schematic structure shown below).

The PEG linked hybrids **3a–c** exhibited lower affinity for p300 – *K*_d_ **3c** = 144(±13) μM, **3a/b** > 200 μM (Fig. 2c) – than FITC-HIF-1α_794–826_ (*K*_d_ = 6.74 ± 0.54 μM).^32^ This suggests either that the linker is not sufficiently long or that the side chains from the linker between helices 2 and 3 make productive non-covalent contacts with p300. A requirement for optimal linker length is supported by the fact that as the length of the PEG linker in **3a–c** increases, so does binding affinity. In spite of the weaker binding affinities observed for **3a–c**, the weak binding for HIF_794–804_ (helix 2) and HIF_816–826_ (helix 3) in isolation and lack of allosteric co-operativity between the two observed previously,^32^ indicates chelate co-operativity for **3a–c** here and validates the approach.

At first the lower inhibitory potency of both HIF-1α_794–826_ and hybrid **2** in comparison to helix mimetic **1** (9.2(±0.9) μM) which we determined previously,^33^ might seem counterintuitive. One explanation that might account for this observation is that there may be a non-specific component to PPI inhibition associated with the aromatic oligoamide helix mimetic **1** (supported in part by a non-unity Hill co-efficient for the curve fitting, a property not observed for **2**). Indeed, more hydrophobic compounds frequently bind with greater potency but poorer specificity and selectivity,^37^ whilst even for peptides, truncation can similarly lead to more potent but less specific binding.^38^ We therefore performed further biophysical analysis. Although we illustrated previously that inhibition of HIF-1α/p300 by **1** was specific to the scaffold and selective in comparison to the eIF4E/4G interaction,^33^ we expanded the range of assays here. Compound **1** exhibited inhibition of the p53/*h*DM2 interaction (IC_50_ = 16.4 ± 1.0 μM) when assessed in a fluorescence anisotropy competition assay (Fig. 3). In contrast, hybrid **2** was shown to exhibit minimal inhibition of this interaction (p53/*h*DM2 IC_50_ > 100 μM). Thus, by adding native amino acid residues from the HIF-1α sequence to the core helix mimetic **1** in hybrid **2**, the specificity and selectivity of binding towards target interactions was improved.

**Fig. 3:**
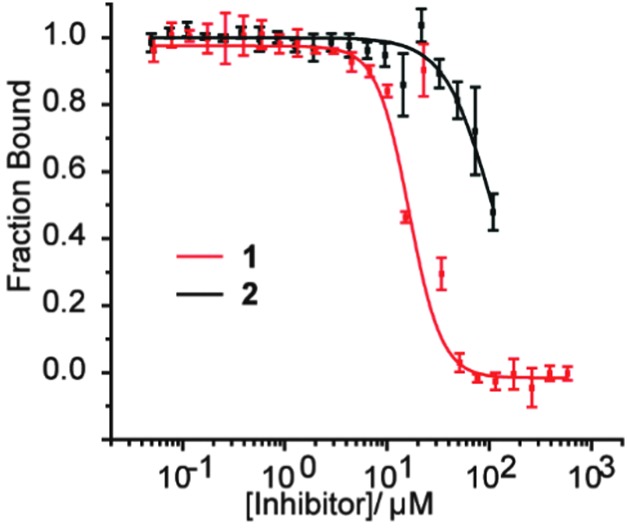
Biophysical screening of helix mimetic **1** and hybrid **2** to profile selectivity; FA competition data for inhibition of p53/hDM2.

In conclusion, we have described the first example of a peptide-helix mimetic hybrid and in doing so conceptually illustrated that extended sequences from proteins can be replaced with molecules that topographically mimic such sequences. Our immediate efforts will focus on structural and biophysical studies on these hybrid mimetics with a view to optimizing the binding properties. Future efforts will focus on incorporating such secondary structure mimetics into longer protein sequences and exploring this replacement strategy for a broader array of protein functions. Application of this generic approach for preparation of functional peptide-helix mimetic hybrids, could allow assembly of protein-like objects with enhanced properties *e.g.* proteolytic and thermal stability, married with superior recognition properties (*e.g.* selectivity) when compared with simpler proteomimetics.

We thank AstraZeneca and EPSRC for PhD studentships (G. M. B. and H. F. K.) and the European Research Council [ERC-StG-240324, and ERC-PoC 632207] for support.
