# Supplementary material for: Towards “bionic” proteins: replacement of continuous sequences from HIF-1α with proteomimetics to create functional p300 binding HIF-1α mimics
Source: Chem Commun (Camb). 2016 Mar 24;52(31):5421–4. doi: 10.1039/c6cc01812b (PMC4843846; doi:10.1039/c6cc01812b)
Supplement: Supplementary file 1 [file CC-052-C6CC01812B-s001.pdf]

Electronic Supplementary Material (ESI) for ChemComm.
This journal is © The Royal Society of Chemistry 2016

Towards "Bionic" Proteins: Replacement of a Helical Sequence with an Aromatic Oligoamide

George M. Burslem,[a, b] Hannah F. Kyle,[b,c] Alex Breeze,[b,c,d] Thomas A. Edwards,[b, c] Stuart L. Warriner,[a, b] Adam Nelson[a, b] and Andrew J. Wilson*[a, b]

Supporting Information

[a] School of Chemistry, University of Leeds, Woodhouse Lane, Leeds LS29JT, UK, [b] Astbury Centre for Structural Molecular Biology, University of Leeds, Woodhouse Lane, Leeds LS29JT, UK, [c] School of Molecular and Cellular Biology, Faculty of Biological Sciences, University of Leeds, Woodhouse Lane, Leeds LS2 9JT, UK, [d] Discovery-Enabling Capabilities and Sciences, AstraZeneca R&D, Alderley Park, Cheshire SK10 4TG, UK.

General Experimental 2
Oligobenzamide Nomenclature 3
Synthesis 4
    Preparation of Peptide-Oligobenzamide Hybrids 8
    Preparation of Peptide-PEG Hybrids 9
Alternative PEG Linking Strategies 11
Binding Assays 14
Spectra 16
References...21

General Experimental

All commercial solvents and reagents were used without further purification unless stated otherwise. All non-aqueous reactions were performed under an atmosphere of nitrogen and using anhydrous solvents. Water-sensitive reactions were performed in oven-dried glassware, cooled under nitrogen before use, or flame dried and cooled, under vacuum if stated. Solvents were removed under reduced pressure using a Büchi rotary evaporator. Ether refers to diethyl ether and petrol refers to petroleum spirit (b.p. 40-60 °C). Flash column chromatography was carried out using silica (35-70 μm particles) or alumina (neutral, Brockman activity 1), with crude reaction mixtures loaded in the initial solvent system or its least polar constituent. Thin layer chromatography was carried out on commercially available silica pre-coated aluminium plates (Kieselgel 60 F254, Merck) or commercially available alumina pre-coated glass plates (neutral, Brockman activity 1). Strong cation exchange columns were carried out using SCX, 5.0 g pre-packed cartridge, Supelco.

Proton and carbon NMR spectra were recorded on a Bruker Avance 500, Avance DPX300 or DRX500 spectrophotometer with an internal deuterium lock. Carbon NMR spectra were recorded with composite pulse decoupling using the waltz 16 pulse sequence. Chemical shifts are quoted in parts per million downfield of tetramethylsilane, and coupling constants (*J*) are given in Hz. NMR spectra were recorded at 300 K unless otherwise stated. Infra-red spectra were recorded using a Perkin–Elmer Spectrum One FT-IR spectrophotometer. Melting points were determined using a Griffin and George melting point apparatus and are uncorrected. Nominal mass spectrometry was routinely performed on a Bruker HCT Ultra spectrometer using electrospray (+) ionization. Nominal and accurate mass spectrometry using electrospray ionisation was carried out by staff or the candidate in the School of Chemistry using a Micromass LCT-KA111, Bruker MicroTOF or Bruker MaXis Impact TOF mass spectrometer. Mass-directed HPLC purifications were run on an Agilent 1260 Infinity Preparative HPLC system equipped with a Waters XBridge™ Prep C18 19 × 100 mm column, 5 μm particle size, on an acetonitrile or methanol/water gradient (5-95% acetonitrile or methanol over 8 minutes) and an Agilent 6120 Quadrupole system equipped with a quadrupole MS detector, using electrospray ionisation (ESI).

Oligobenzamide Nomenclature

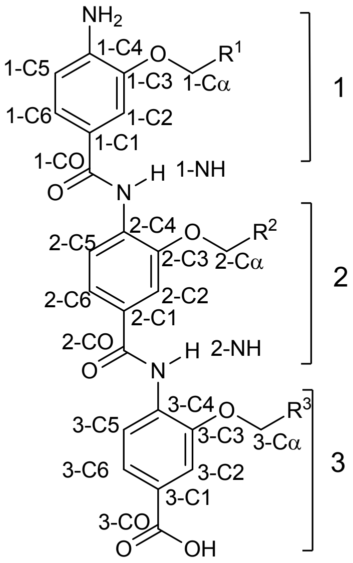

To simplify the numbering and NMR assignment of oligobenzamides, we have devised a sequential nomenclature, where each of the monomer building blocks is considered separately. The monomers are numbered from 1 to 3 starting from the *N*-terminal. Within each monomer, the numbering is the same: the carbons from the aminobenzoic acid are numbered using the standard system (the aromatic carbon bearing the carboxylic acid is C1, the one bearing the amine is C4). Then, the lateral chain is numbered: the carbon attached to the oxygen is the Cα, and the numbering of the aliphatic part of the side chain continues with Cβ, etc. In the case of aromatic side chains, the aromatic carbons are numbered CAr1, CAr2, etc. The numbering of the protons is based on the carbon numbering. To differentiate each individual carbon/proton, the monomer number is added as a prefix to the carbon/proton number representative examples are given above.

Synthesis

Prop-2'-en-1'-yl 3-hydroxy-4-nitrobenzoate, 3

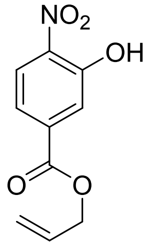

Para-toluene sulfonic acid (2 g, 11 mmol) was added to a solution of 3-hydroxy-4-nitrobenzoic acid (10 g, 55 mmol) in allyl alcohol (30 ml) and the reaction heated to 80 °C overnight then allowed to cool to r.t. The reaction mixture was diluted with ethyl acetate (50 ml) and washed with water (30 ml), saturated aqueous sodium bicarbonate (2 × 30 ml) and brine (30 ml), dried over $MgSO_4$ and concentrated *in vacuo* to a yellow amorphous solid (8.9 g, 73%) ν_{max}/cm^{-1} (solid state) 3316, 1722; ^1H NMR (500 MHz, $CDCl_3$) δ 10.53 (s, 1H, phenol OH), 8.20 (d, *J* = 8.8 Hz, 1H, 6-CH), 7.87 (s, 1H, 2-CH), 7.66 (d, *J* = 8.8 Hz, 1H, 5-CH), 6.06 (ddd, *J* = 16.7, 11.2, 5.6 Hz, 1H, 2'C-H), 5.46 (d, *J* = 17.2 Hz, 1H, 3'C-H_{trans}), 5.36 (d, *J* = 10.4 Hz, 1H, 3'C-H_{cis}), 4.88 (d, *J* = 5.6 Hz, 2H, 1'-CH_2); ^{13}C NMR (125 MHz, $CDCl_3$) δ 164.01, 154.67, 138.05, 135.83, 131.43, 125.29, 121.71, 120.65, 119.22, 66.55; HRMS Found 222.0411 $C_{10}H_9NO_5$ $[M-H]^-$ requires 222.0407

Prop-2'-en-1'-yl 3-(2''-methylpropoxy)-4-nitrobenzoate, 4

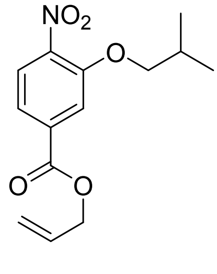

1-bromo-2-methylpropane (3.6 ml, 33.6 mmol) was added to a suspension of Prop-2'-en-1'-yl 3-hydroxy-4-nitrobenzoate (5 g, 22.4 mmol) and potassium carbonate (15.4 g, 112 mmol) in dimethylformamide (50 ml) and the reaction heated to 50 °C with stirring overnight. The reaction mixture was then diluted with ethyl acetate (100 ml), washed copiously with water and brine, dried over $MgSO_4$ and concentrated *in vacuo* to give the title compound as a red oil (3.55 g, 60%). ν_{max}/cm^{-1} (solid state) 2961, 2875, 1718; ^1H NMR (500 MHz, $CDCl_3$) δ 7.80 (d, *J* = 8.4 Hz, 1H, 5-CH), 7.73 (s, 1H, 2C-H), 7.67 (dd, *J* = 8.4, 1.3 Hz, 1H, 6-CH), 6.04 (ddd, *J* = 16.3, 11.0, 5.8 Hz, 1H, 2'-CH), 5.43 (dd, *J* = 17.2, 1.2 Hz, 1H, 3'-CH_{trans}), 5.33 (dd, *J* = 10.3, 0.8 Hz, 1H, 3'-CH_{cis}), 4.86 (d, *J* = 5.8 Hz, 2H, 1'-CH_2), 3.93 (d, *J* = 6.4 Hz, 2H, 1''-CH_2), 2.15 (spt, *J* = 6.6 Hz, 1H, 2''-CH), 1.06 (d, *J* = 6.9 Hz, 6H, 3''-CH_3 and 4''CH_3); ^{13}C NMR (125 MHz, $CDCl_3$) δ 164.45, 152.05, 142.51, 134.75, 131.64, 125.15, 121.06, 118.98,

115.42, 75.95, 66.39, 28.19, 18.98; HRMS Found: 302.0998, $C_{14}H_{17}NO_5$ $[M+Na]^+$ requires 302.0998.

Prop-2'-en-1'-yl 4-amino-3-(2''-methylpropoxy)benzoate, 5

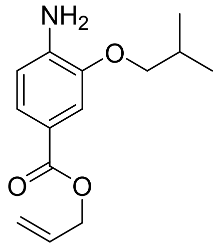

Tin (II) chloride dihydrate (4 g, 17.9 mmol) was added to a solution of prop-2'-en-1'-yl 3-(2''-methylpropoxy)-4-nitrobenzoate (1 g, 3.58 mmol) in ethyl acetate (70 ml) and the reaction heated to 50 °C overnight. The reaction was allowed to cool and poured into 1M NaOH solution (50 ml). The organic layer was separated and washed with 1M NaOH (3 × 30 ml), dried over $MgSO_4$ and concentrated *in vacuo* to a pale yellow oil (880 mg, 98%). ν_{max}/cm^{-1} (solid state) 3588, 2914, 1742, 1635; ^1H NMR (500 MHz, $CDCl_3$) δ 7.60 (dd, *J* = 8.2, 1.6 Hz, 1H, 6-CH), 7.48 (d, *J* = 1.4 Hz, 1H, 2C-H), 6.69 (d, *J* = 8.2 Hz, 1H, 5-CH), 6.07 (ddd, *J* = 17.2, 10.4, 1.4 Hz, 1H, 2'-CH), 5.42 (dd, *J* = 17.2, 1.4 Hz, 1H, 3'-CH_{trans}), 5.29 (dd, *J* = 10.4, 1.4 Hz, 1H, 3'-CH_{cis}), 4.81 (d, *J* = 5.6 Hz, 2H, 1'-CH_2), 3.85 (d, *J* = 6.5 Hz, 2H, 1''-CH_2), 2.25 – 1.98 (sept, 6.6 Hz, 1H, , 2''-CH), 1.08 (d, *J* = 6.6 Hz, 6H, 3''-CH_3 and 4''CH_3); ^{13}C NMR (125 MHz, $CDCl_3$) δ 166.54, 145.60, 141.36, 132.82, 123.99, 119.40, 117.70, 113.08, 112.15, 74.72, 65.07, 28.30, 19.34; HRMS Found: 250.1438, $C_{14}H_{19}NO_3$ $[M+H]^+$ requires 250.1437

Prop-2'-en-1'-yl4-(2-{[(9H-fluoren-9-ylmethoxy)carbonyl]amino}acetamido)-3-(2''-methylpropoxy)benzoate, 6

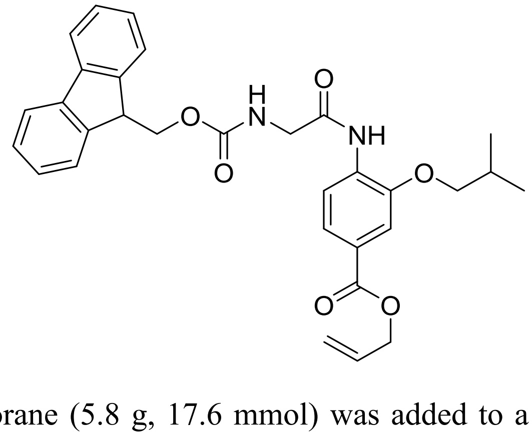

Dichlorotriphenylphosphorane (5.8 g, 17.6 mmol) was added to a solution of Fmoc-glycine (2 g, 7.06 mmol) and prop-2'-en-1'-yl 4-amino-3-(2''-methylpropoxy)benzoate (880 mg, 3.53 mmol) in chloroform (50 ml) and the reaction heated to reflux overnight. The reaction was allowed to cool to r.t. and concentrated *in vacuo*. The residue was purified by column chromatography eluting with dichloromethane to give the *title compound* as a colourless amorphous solid (1.3g, 71%). ν_{max}/cm^{-1} (solid state) 3398, 2958, 2873, 1697; ^1H NMR (500

MHz, $CDCl_3$) δ 8.59 (s, 1H, Amide NH), 8.48 (d, *J* = 8.4 Hz, 1H, Ar-H), 7.80 (d, *J* = 6.3 Hz, 1H, Ar-H), 7.74 (d, *J* = 8.4 Hz, 1H, Ar-H), 7.62 (s, 1H, Ar-H), 7.56 (d, *J* = 1.2 Hz, 1H, Ar-H), 7.43 (s, 1H, Ar-H), 7.33 (s, 1H, Ar-H), 6.08 (ddd, *J* = 17.2, 10.4, 5.6 Hz, 1H, 2'-CH), 5.44 (d, *J* = 17.2 Hz, 1H, 3'-CH_{trans}), 5.33 (d, *J* = 10.4 Hz, 1H, 3'-CH_{cis}), 4.85 (d, *J* = 5.6 Hz, 2H, 1'-CH_2), 4.48 (d, *J* = 7.0 Hz, 2H, gly-CH_2), 4.26 (t, *J* = 6.9 Hz, 1H, Fmoc-CH), 4.10 (s, 1H br s, 1H, carbamate NH), 3.85 (d, *J* = 6.4 Hz, 2H, Fmoc-CH_2), 2.12 (sept, *J* = 6.7 Hz, 1H, 2''-CH), 1.04 (d, *J* = 6.7 Hz, 6H, 3''-CH_3 and 4''CH_3); ^{13}C NMR (125 MHz, $CDCl_3$) δ 167.19, 165.92, 146.97, 143.62, 141.33, 132.32, 131.40, 127.84, 127.12, 125.49, 124.99, 123.21, 120.07, 118.67, 118.28, 111.66, 75.15, 67.58, 65.63, 47.08, 45.90, 28.20, 19.25; HRMS found: 529.234309, $C_{31}H_{32}N_2O_6$ $[M+H]^+$ Requires 529.233313.

4-(2-{[(9H-fluoren-9-ylmethoxy)carbonyl]amino}acetamido)-3-(2-methylpropoxy)benzoic acid, 7

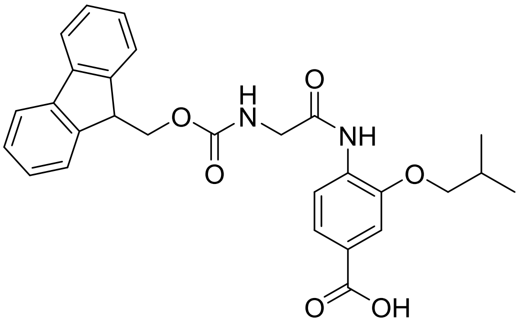

Palladium tetrakis(triphenylphosphine) (110 mg, 0.095 mmol, 5 mol%) was added to a solution of Prop-2'-en-1'-yl4-(2-{[(9H-fluoren-9-ylmethoxy)carbonyl]amino}acetamido)-3-(2''-methylpropoxy)benzoate (1 g, 1.89 mmol) and sodium *p*-toluenesulfinate (505 mg, 2.83 mmol) in tetrahydrofuran (50 ml) and the reaction stirred at r.t. for 24 hours. The reaction mixture was filtered through Celite and concentrated *in vacuo*. The residue was purified by column chromatography eluting with 5-10% methanol in dichloromethane to give the *title compound* as a yellow solid (741 mg, 80%). ν_{max}/cm^{-1} (solid state) 3395, 3302, 2963, 1697; ^1H NMR (500 MHz, d_6-DMSO) δ 8.98 (s, 1H, Amide N-H) 8.24 (d, *J* = 8.0 Hz, 1H, Ar-H), 7.95 (br. s, 1H, carbamate-NH), 7.90 (d, *J* = 7.4 Hz, 2H, Ar-H), 7.72 (d, *J* = 7.4 Hz, 2H, Ar-H), 7.57 (d, *J* = 7.4 Hz, 1H, Ar-H), 7.52 (s, 1H, Ar-H), 7.42 (t, *J* = 7.2 Hz, 2H, Ar-H), 7.33 (t, *J* = 7.3 Hz, 2H, Ar-H), 4.34 (d, *J* = 6.6 Hz, 2H, Fmoc-CH_2), 4.25 (t, *J* = 6.6 Hz, 1H, Fmoc-CH), 3.86 (d, *J* = 6.0 Hz, 2H, Gly-CH_2), 3.83 (d, *J* = 6.6 Hz, 2H, 1"-CH_2), 2.07 – 1.94 (m, 1H, 2''-CH), 0.95 (d, *J* = 6.7 Hz, 6H, 3''-CH_3 and 4''CH_3);^{13}C NMR (125 MHz, d_6-DMSO) δ 168.27, 147.24, 143.68, 140.72, 130.81, 127.65, 127.05, 125.14, 122.29, 120.11, 118.80, 112.00, 74.62, 66.01, 46.57, 27.59, 18.94; HRMS found 489.2032 $C_{28}H_{28}N_2O_6$ requires $[M+H]^+$ 489.2020

Preparation of Peptide-Oligobenzamide Hybrid, 2

Prepared following an adapted literature method.[1] Fmoc-monomer acid chlorides were prepared as previously described and loaded onto a CEM liberty peptide synthesiser. Gly-loaded Wang resin was swelled in NMP for 30 minutes prior to loading onto the synthesiser. The previously described methods were used to synthesise the oligobenzamide portion of the molecule, ending with the Fmoc-Gly-monomer. The peptide portion was then extended using standard Fmoc peptide synthesis.

Ac-QLTSYSEVNAPIQSRNLLQG--[*O*-^{i}Bu(3-HABA)]-[*O*-^{i}Bu(3-HABA)]-[*O*-^{i}Pr(3-HABA)]-G-NH$_2$, 138

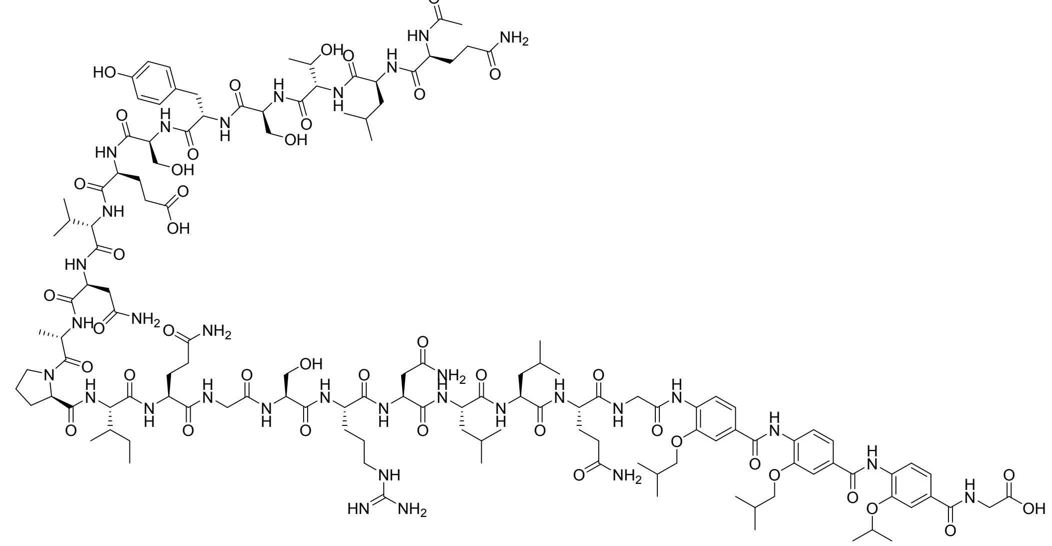

LC-MS *m/z* (ES) 1468.2 $[M+2H]^{2+}$ HRMS Found: 1467.7340, $C_{133}H_{201}N_{33}O_{42}$ requires $[M+2H]^{2+}$ 1467.7393

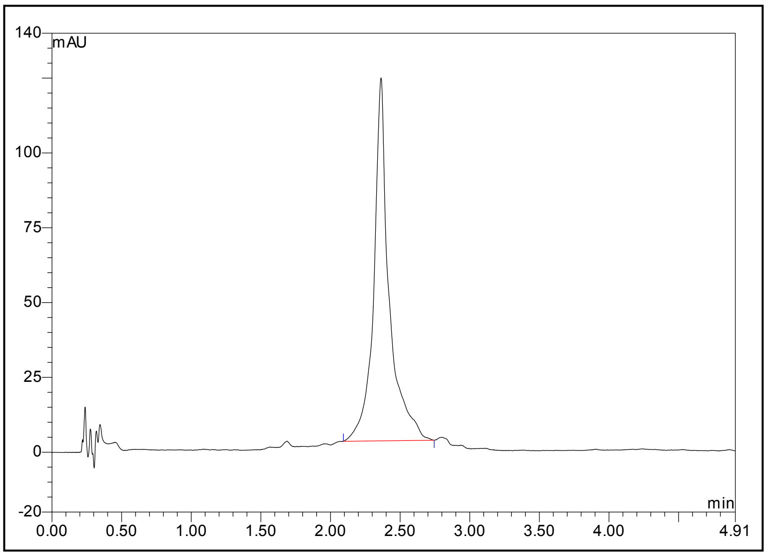

Preparation of Peptide-PEG Hybrids

Fmoc-Amino-PEG$_{8/12/24}$-COOH

Amino-PEG$_{8/12/24}$-COOH (100 mg) was dissolved in 1:1 water/dioxane (6 ml) and sodium carbonate (3 eq.) was added followed by Fmoc-Cl (1.2 eq) and the reaction stirred overnight. The reaction mixture was then acidified with conc. HCl and extracted with ethyl acetate (3 × 5 ml). The combined organic layers were dried over $MgSO_4$ and concentrated *in vacuo*. The product was checked by LC-MS and used without further purification.

Fmoc-Amino-PEG$_8$-COOH

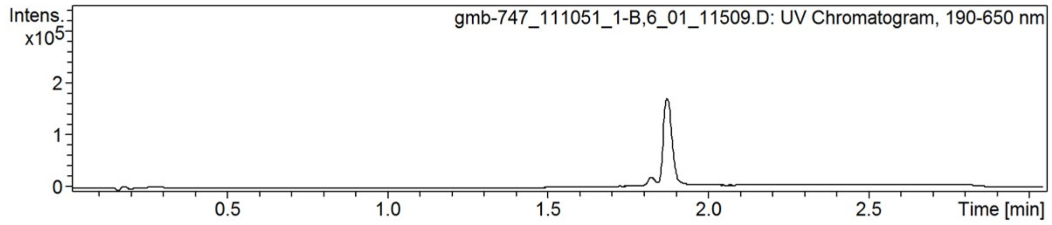

LC-MS *m/z* (ES) 664.5 [M+H]$^+$

Fmoc-Amino-PEG$_{12}$-COOH

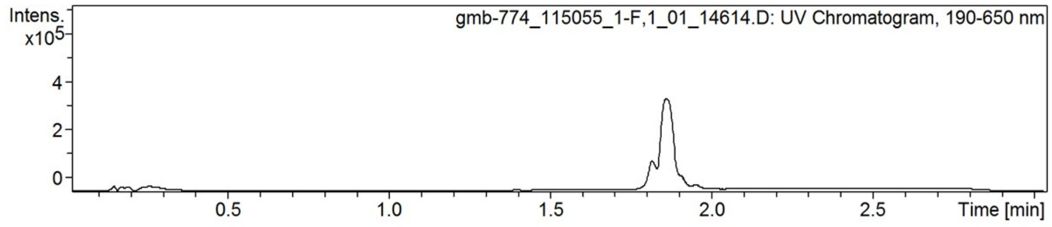

LC-MS *m/z* (ES) 840.7 [M+H]$^+$

Fmoc-Amino-PEG$_{24}$-COOH

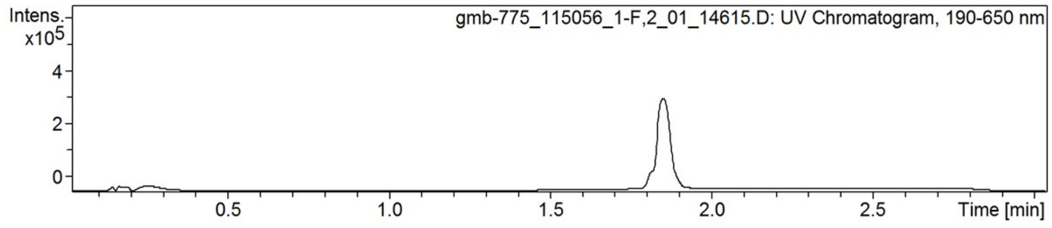

LC-MS *m/z* (ES) 1391.1 [M+Na]$^+$

Peptide-PEG Conjugates

The *C*-terminal portion of the peptide was synthesised using standard Fmoc-SPPS as above on 0.3 mmol scale and checked by cleaving a small amount and performing LC-MS analysis. The resin was then split into 3 equal portions.

To each portion was added the Fmoc-Amino-PEG-COOH monomer (PEG_8 – 2 eq., PEG_{12} – 1.6 eq. and PEG_{24} – 1.1 eq.), HCTU (5 eq.) and DIPEA (5 eq.) in DMF (2 ml). The reaction mixture was agitated for 16 hours and drained. The resin was washed 5 × 2 minutes with DMF and the remainder of the peptide synthesised using standard Fmoc-SPPS.

FITC-Ahx-QLTSYDCEVAN-PEG$_8$-EELLRALDQVN
LC-MS *m/z* (ES) 1150.7 $[M+3H]^{3+}$, 872 $[M+4H]^{4+}$ HRMS Found: 1724.2922
$C_{152}H_{231}N_{33}O_{54}S_2$ requires $[M+2H]^{2+}$ 1724.2922

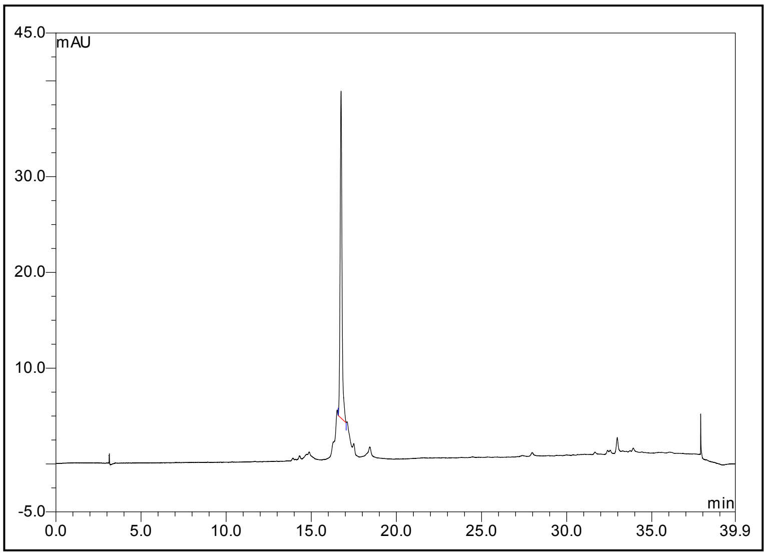

FITC-Ahx-QLTSYDCEVAN-PEG$_{12}$-EELLRALDQVN

LC-MS *m/z* (ES) 1813.1 $[M+2H]^{2+}$, 1209 $[M+3H]^{3+}$ HRMS Found: 1813.3517
$C_{160}H_{247}N_{33}O_{58}S_2$ requires $[M+2H]^{2+}$ 1813.3411

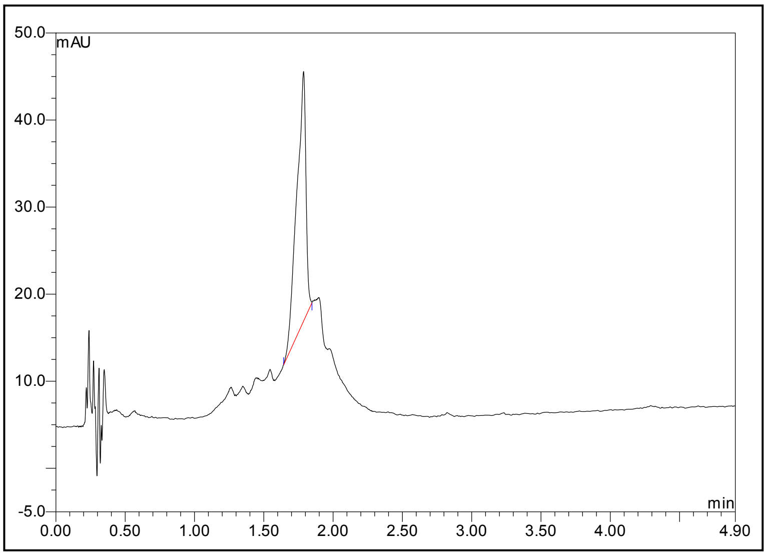

FITC-Ahx-QLTSYDCEVAN-PEG$_{24}$-EELLRALDQVN
LC-MS *m/z* (ES) 1385.5 $[M+3H]^{3+}$, 1039.5 $[M+4H]^{4+}$ HRMS Found: 2077.4836 $C_{184}H_{295}N_{33}O_{70}S_2$ requires $[M+2H]^{2+}$ 2077.4984

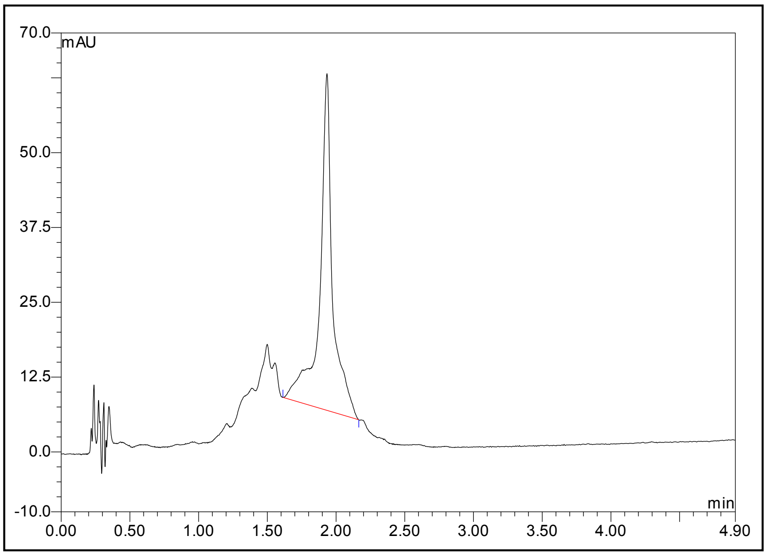

Alternative PEG Linking Strategies

Initially, a one pot conjugation procedure was developed to allow the modular assembly of peptidic regions with linkers of various lengths thus negating the requirement to perform the peptide synthesis in triplicate. A route combining copper catalysed azide alkyne cycloaddition and thiol-ene reactions was envisioned. This was trialled with a model system as shown below. This reaction sequence progressed successfully in one pot as followed by LC-MS. By treating the commercially available NHS-PEG-Maleimide compound with a short azido-alkylamine followed by a thiol, the click handle and the first component can be introduced regioselectively. Finally, addition of an alkyne along with a copper catalyst affords the PEG linked compound.

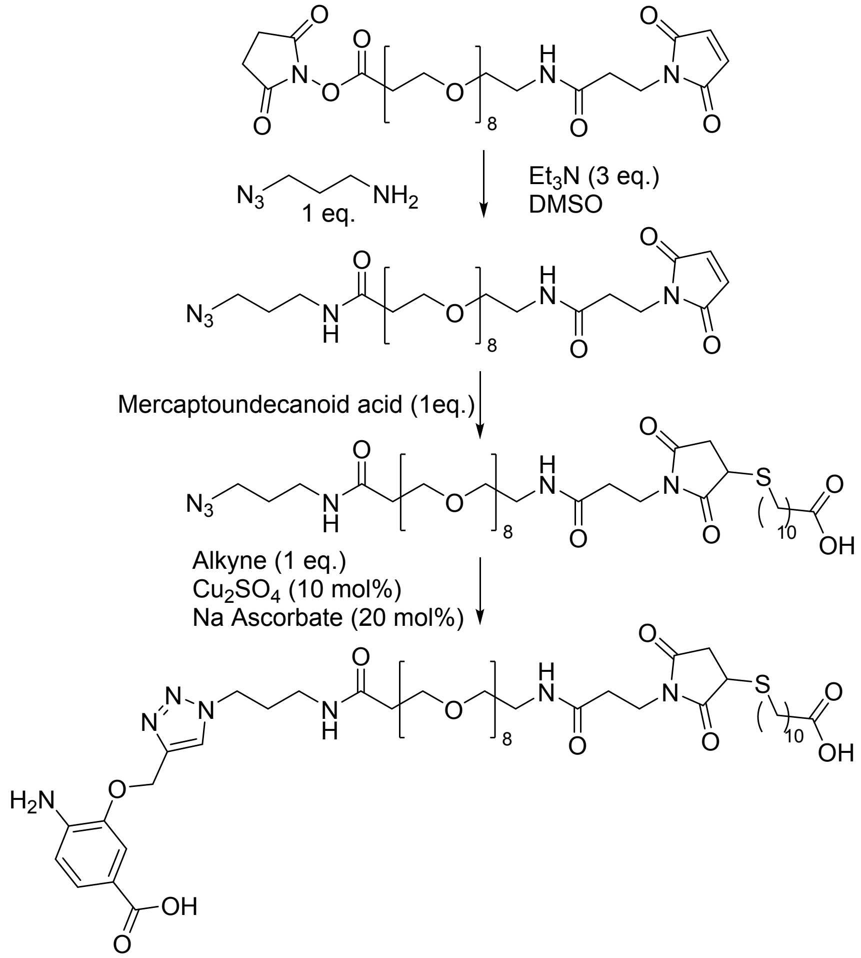

This one pot procedure was then progressed onto using a peptide containing and *C*-terminal propargyl glycine and another with an *N*-terminal thiol as shown below and monitored by LC-MS. The final click reaction progressed with small molecule alkynes but not the alkynyl peptide. The peptide appeared to be unstable to the reaction conditions.

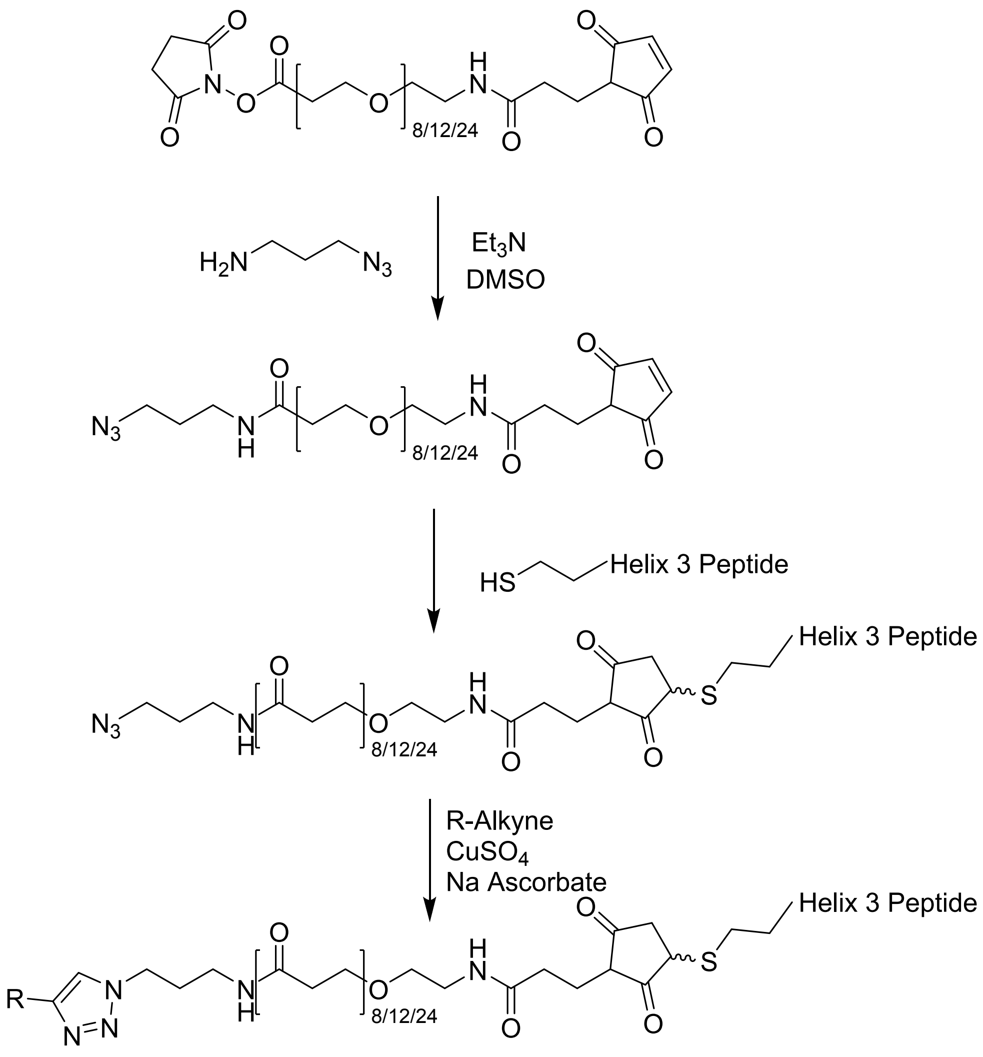

A shorter alkynyl peptide was prepared and used to optimise the click reaction as shown in the table below. Using these optimised conditions, the full length peptide could cleanly undergo click reactions. However, when the complete one pot procedure was attempted using the optimised conditions, no product was observed.

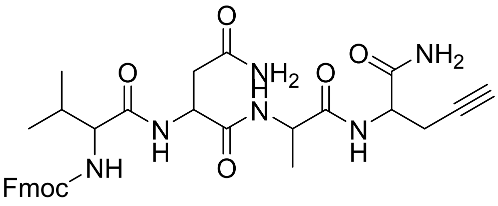

Conditions	Outcome
1 eq. Cu, 1 eq. Ascorbate, DMSO	No Reaction
1.1 eq. Cu, 1 eq. Ascorbate, DMSO	Conversion to product and degradation
1.1 eq. Cu, 10 eq. Ascorbate, DMSO	Conversion to product and degradation
1 eq. Cu, 1 eq. TBTA, 10 eq. Ascorbate, DMSO	Conversion to product and degradation
1.1 eq. Cu, 10 eq. TCEP, DMSO	No Reaction
1 eq. Cu, 10 eq. Ascorbate, 1:1 THF/Water	Conversion to product
1 eq. Cu, 10 eq. Ascorbate, 10 eq. TBTA, 1:1 THF/Water	Conversion to product and stable o/n

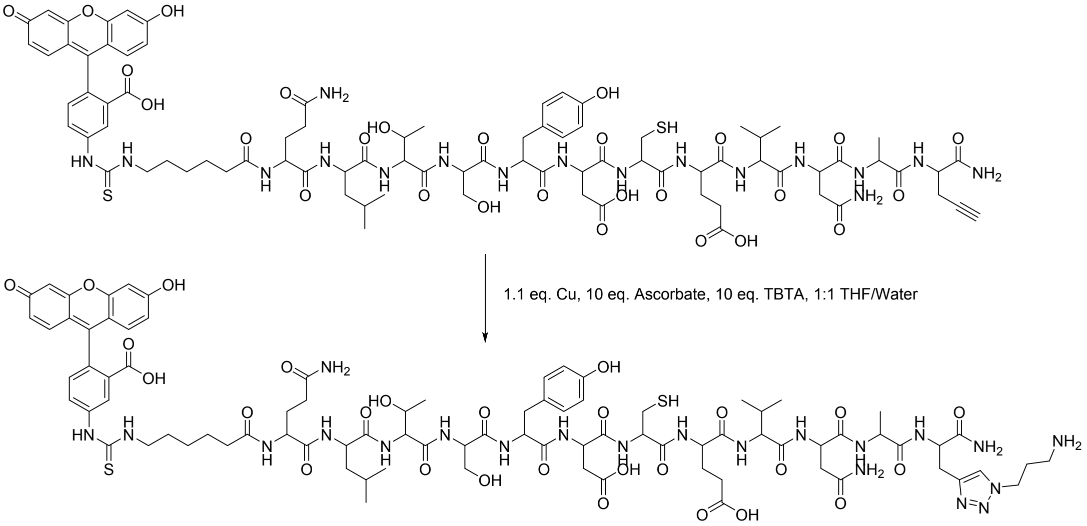

Clean progression by LC-MS

However, when the complete one pot procedure was attempted using the optimised conditions, no product was observed. At this stage it was decided to prepare the PEG-Peptide conjugates via linear peptide synthesis incorporating the PEG chain as with any other monomer.

Binding Assays

Binding assays and protein expression were carried out as previously reported.[2, 3]

The helix 2-3 peptide and the helix 2-extended peptide were purchased from Proteogenix, France and the sequences shown below.

Helix 2-3: Ac-QLTSYDCEVNAPIQGSRNLLQGEELLRALDQVN-NH$_2$

Helix 2-extended: Ac-QLTSYDCEVNAPIQGSRNLLQ-NH$_2$

The competition assay data for the Helix 2-extended peptide is shown below.

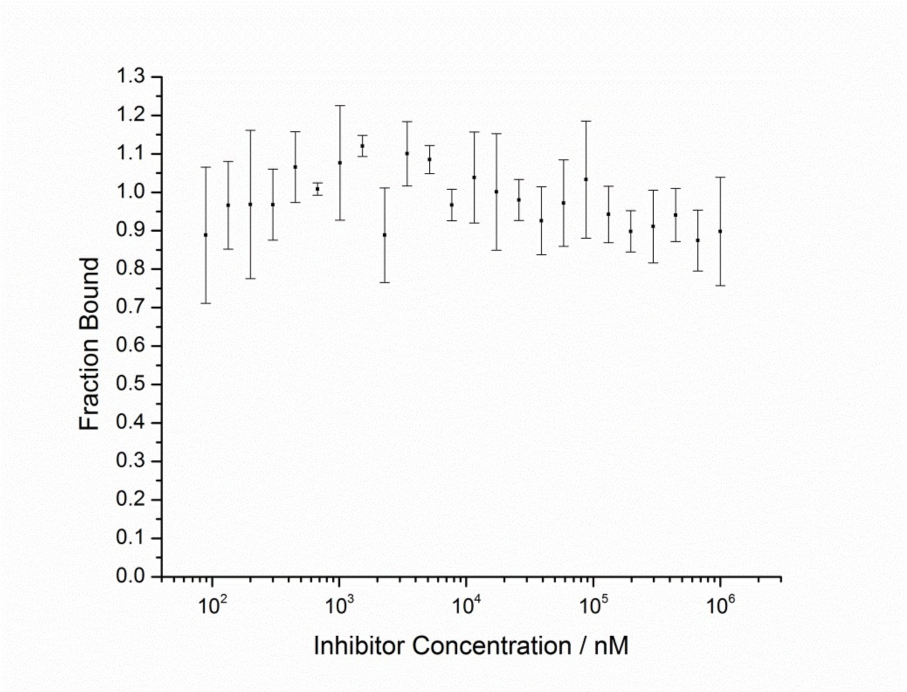

Spectra

Prop-2’-en-1’-yl 3-hydroxy-4-nitrobenzoate, 3

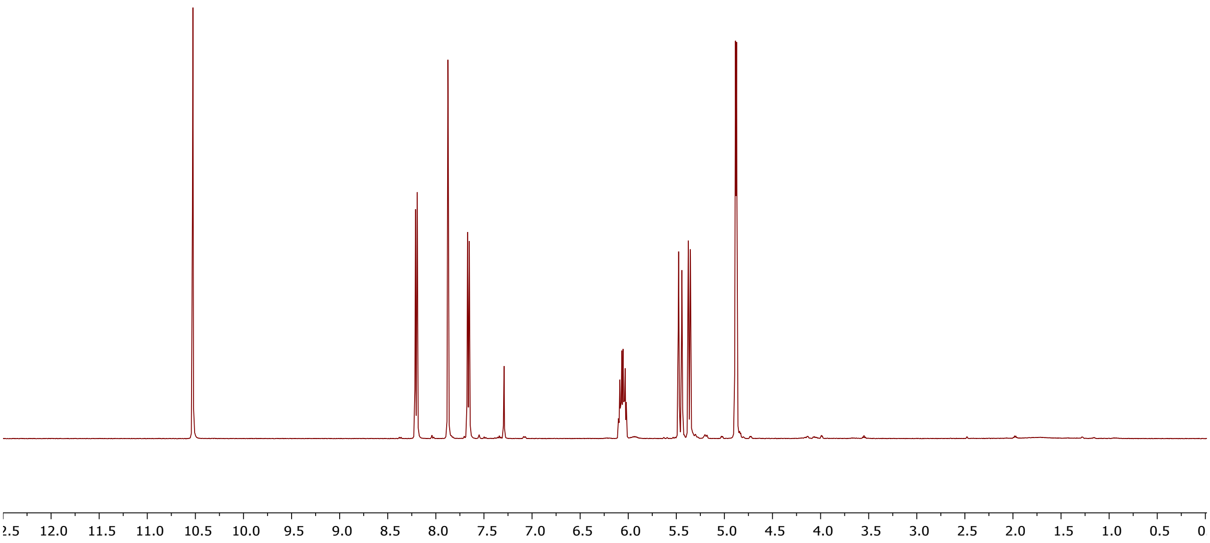

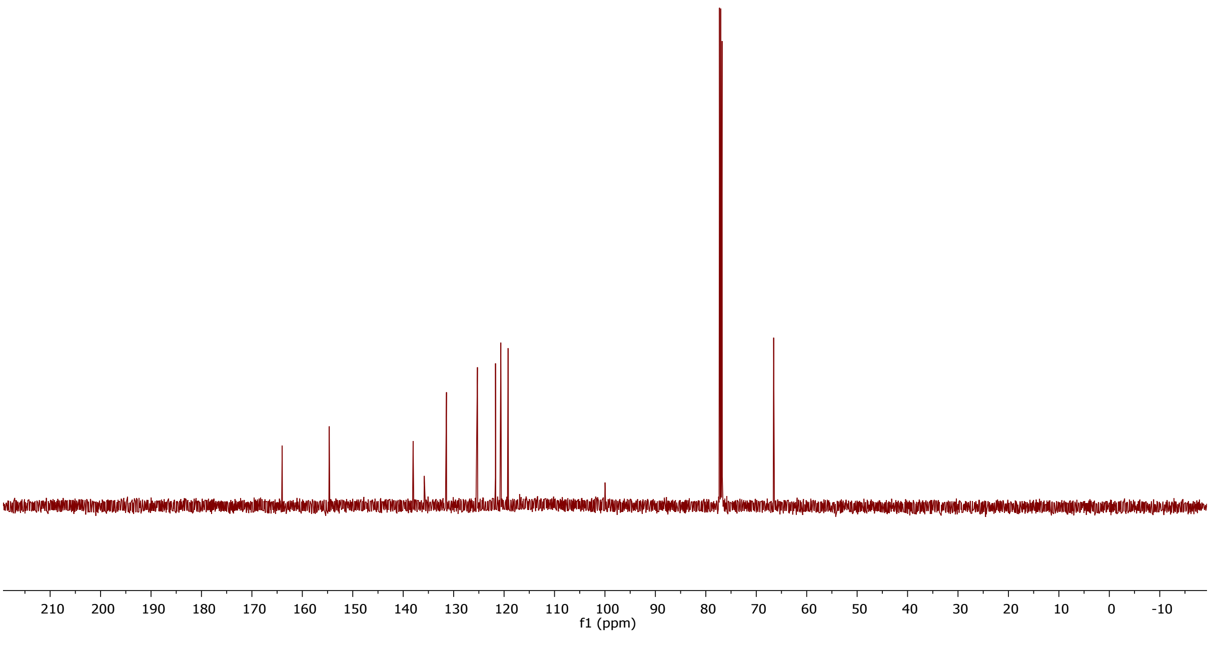

Prop-2’-en-1’-yl 3-(2’’-methylpropoxy)-4-nitrobenzoate, 4

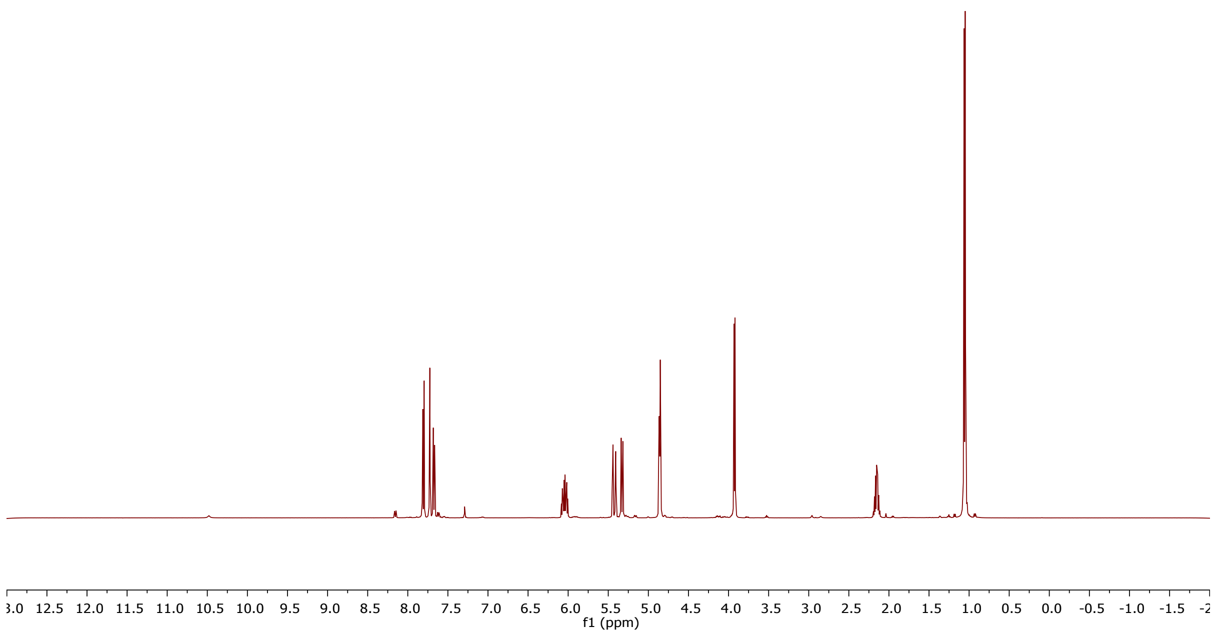

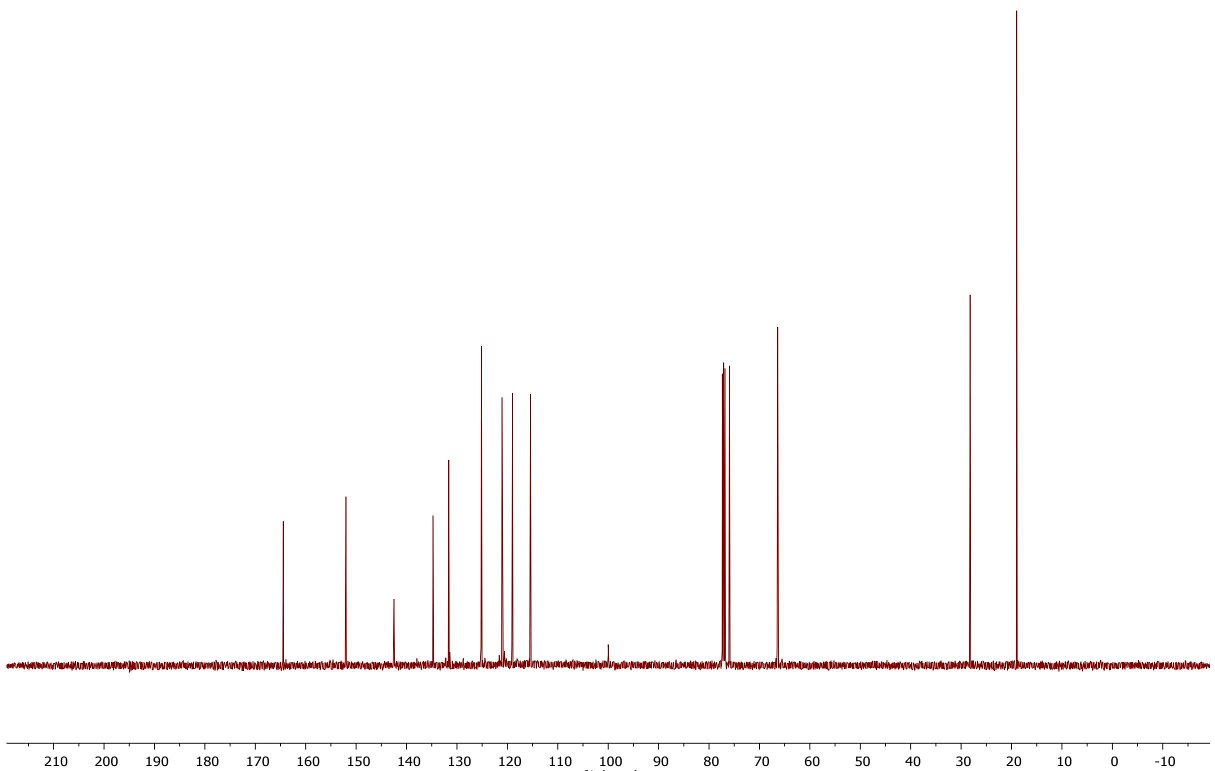

Prop-2’-en-1’-yl 4-amino-3-(2’’-methylpropoxy)benzoate, 5

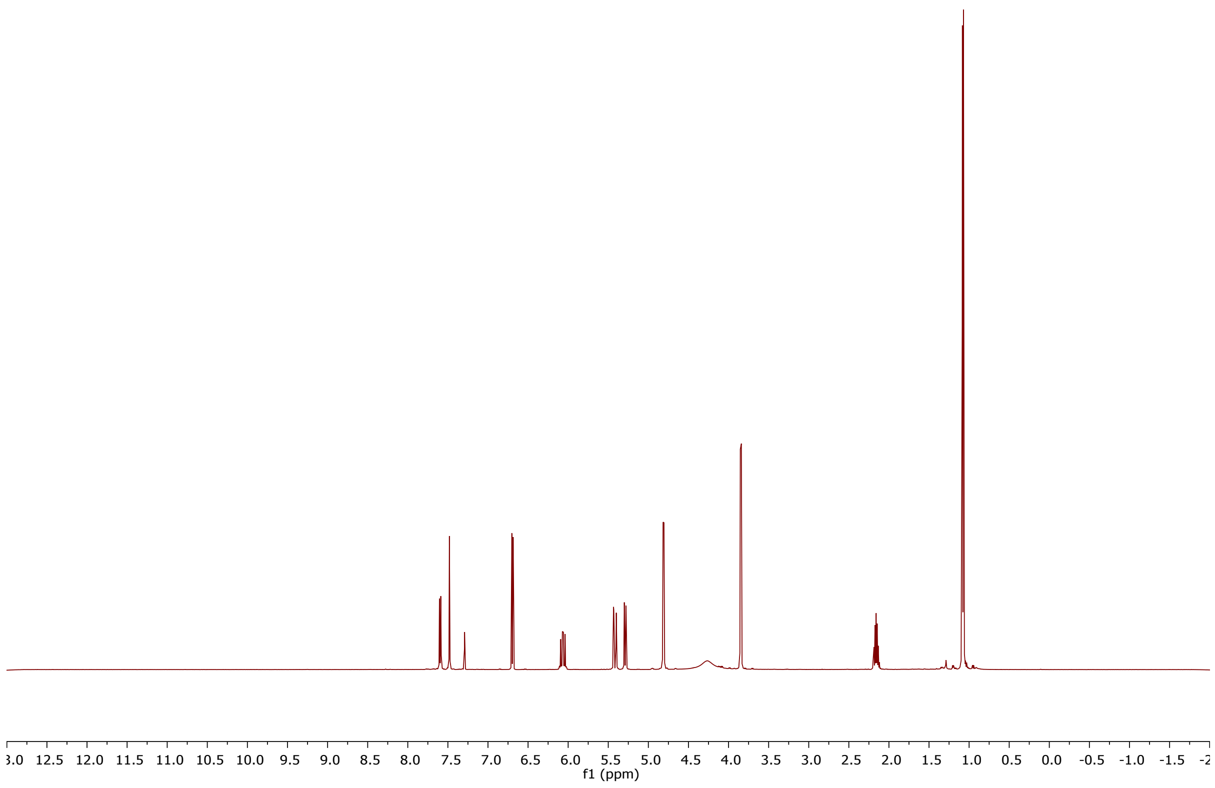

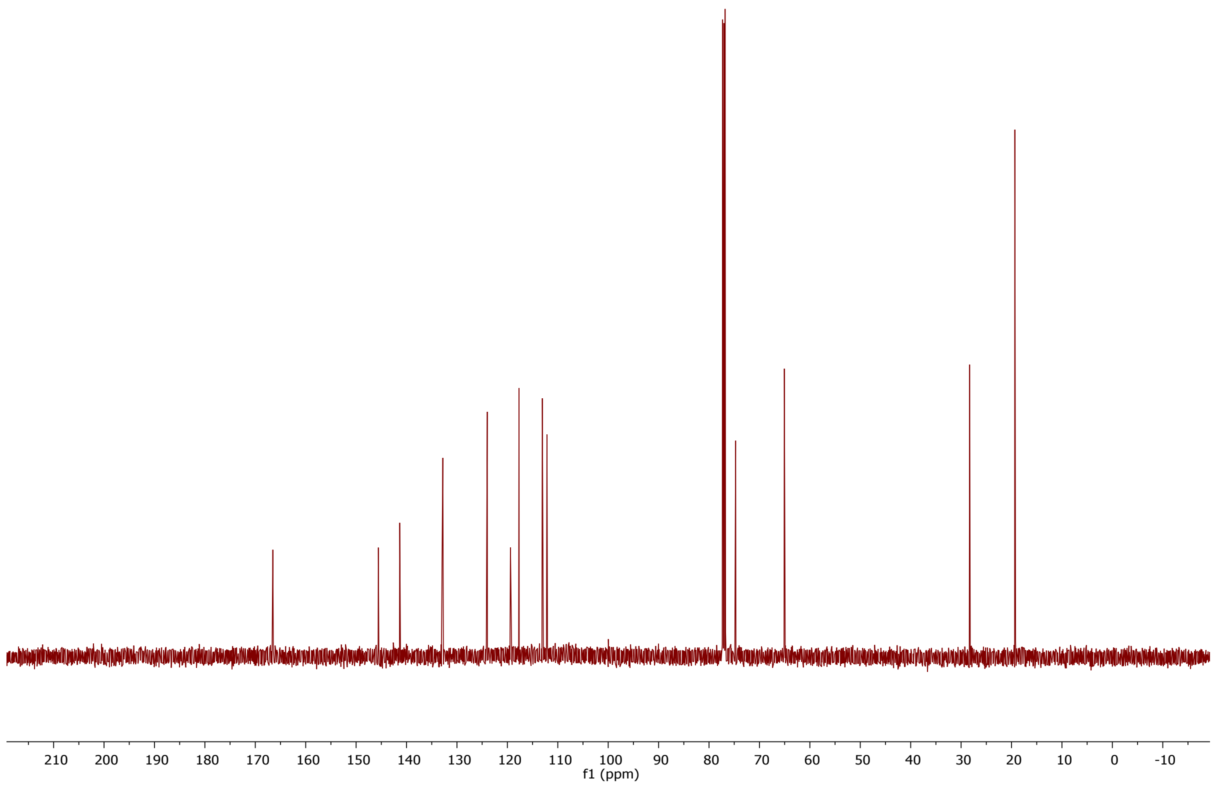

Prop-2’-en-1’-yl4-(2-{[(9H-fluoren-9-ylmethoxy)carbonyl]amino}acetamido)-3-(2’’-methylpropoxy)benzoate, 6

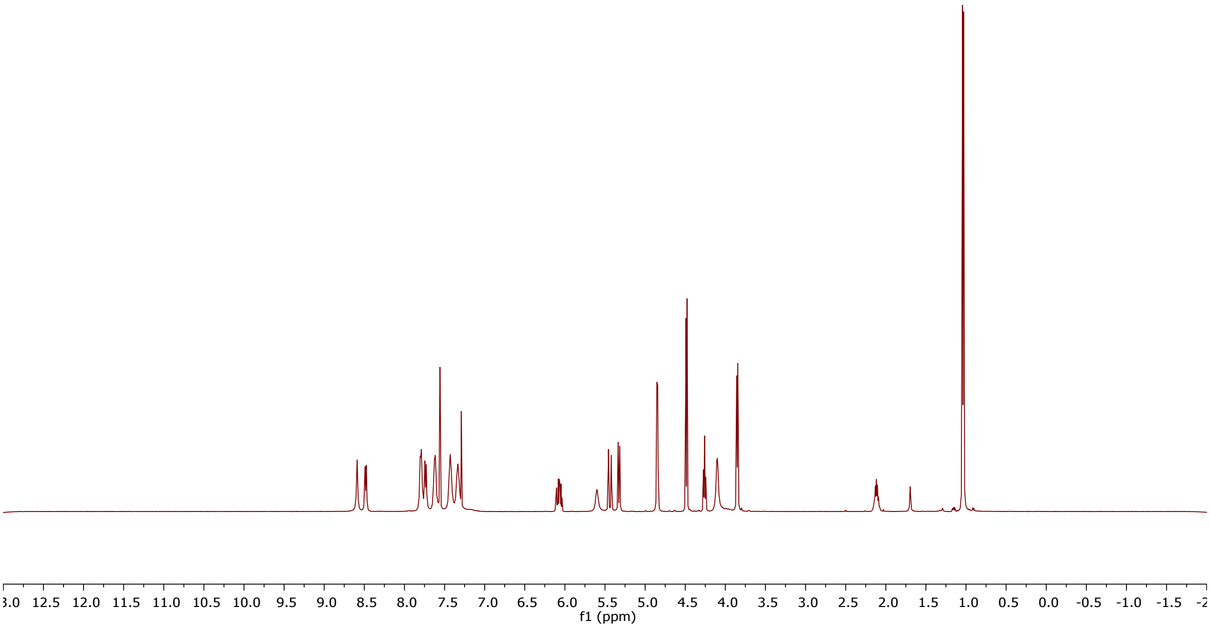

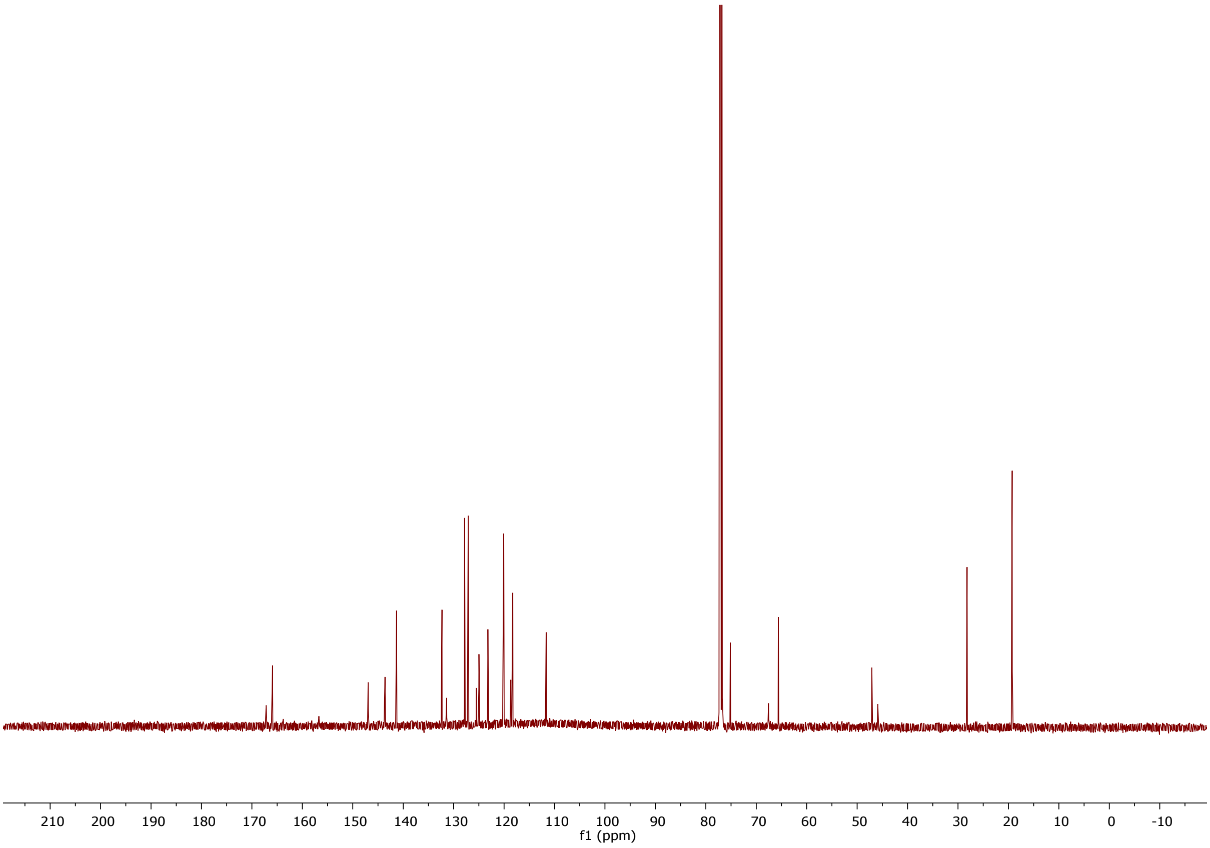

4-(2-{[(9H-fluoren-9-ylmethoxy)carbonyl]amino}acetamido)-3-(2-methylpropoxy)benzoic acid, 7

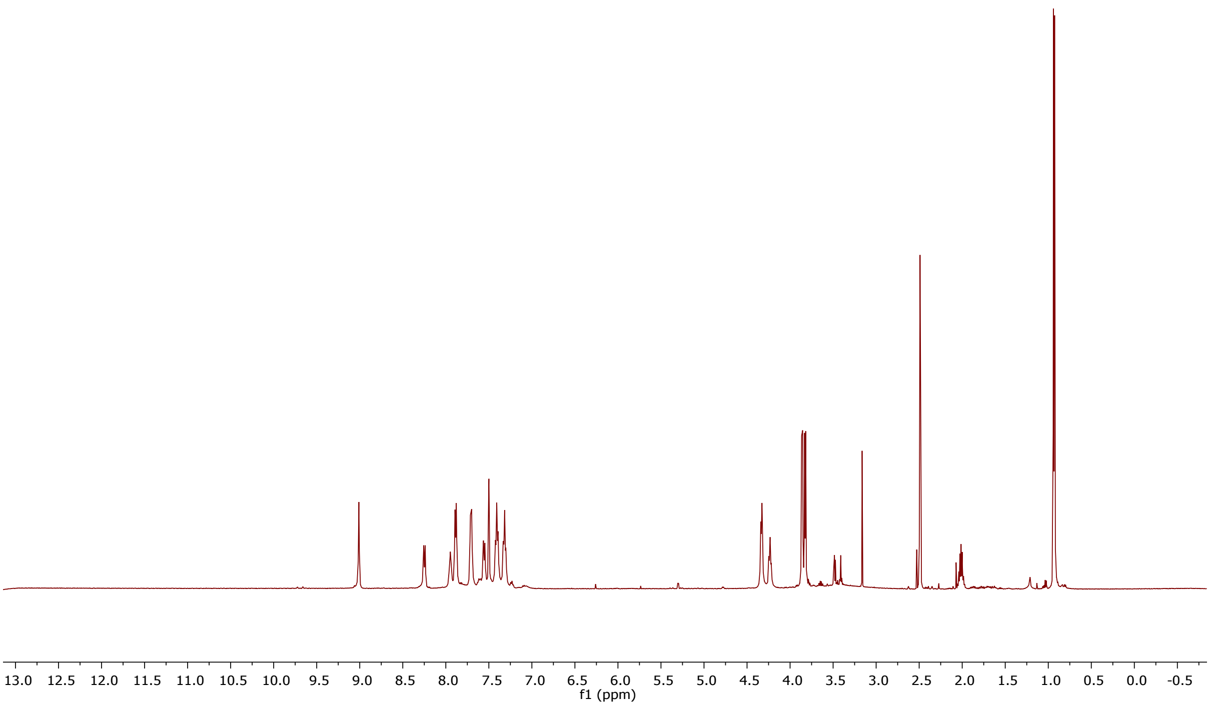

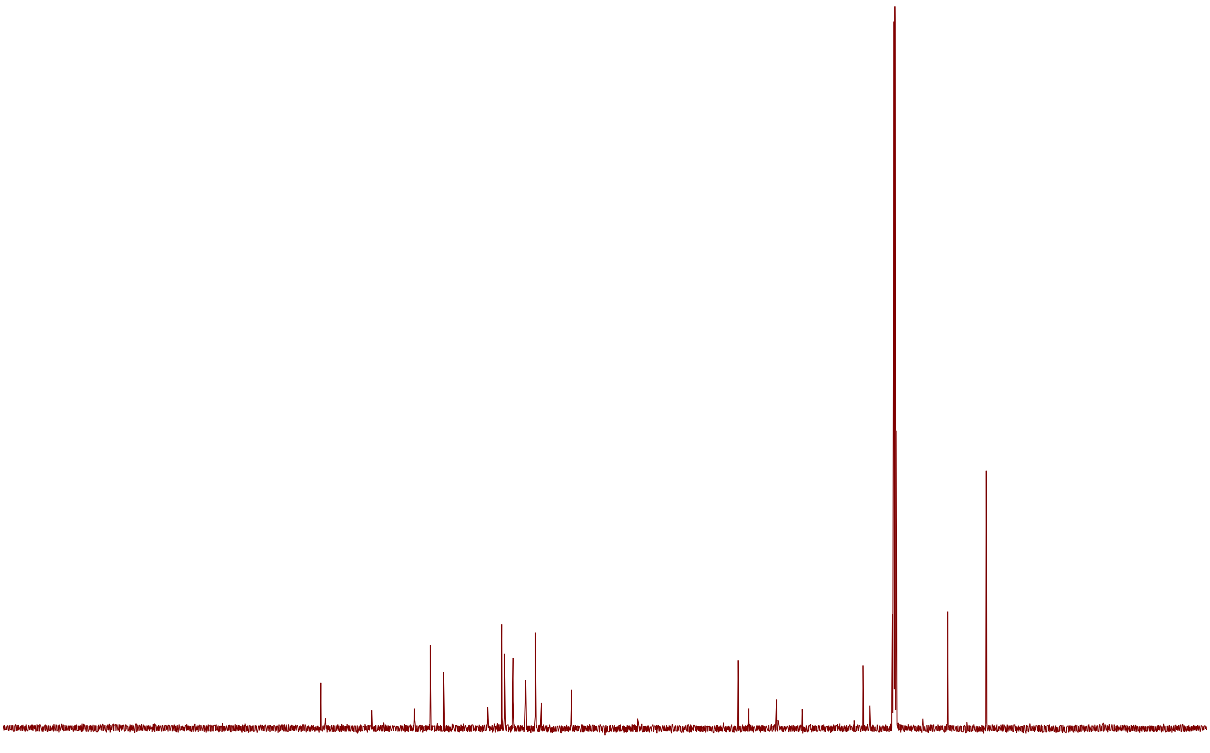

References

1. N. S. Murphy, P. Prabhakaran, V. Azzarito, J. P. Plante, M. J. Hardie, C. A. Kilner, S. L. Warriner and A. J. Wilson, *Chem. Eur. J.*, 2013, **19**, 5546-5550.
2. H. F. Kyle, K. F. Wickson, J. Stott, G. Burslem, A. L. Breeze, C. Tiede, D. C. Tomlinson, S. Warriner, A. Nelson, A. Wilson and T. A. Edwards, *Molecular BioSystems*, 2015.
3. G. M. Burslem, H. F. Kyle, A. L. Breeze, T. A. Edwards, A. Nelson, S. L. Warriner and A. J. Wilson, *ChemBioChem*, 2014, **15**, 1083-1087.
